# Recruiting for Qualitative Studies on Cancer and Multimorbidity in Puerto Rico

**DOI:** 10.1093/geroni/igaf122.1098

**Published:** 2025-12-31

**Authors:** Maira Castañeda-Avila, Karen Ortiz-Ortiz, Kate Lapane

**Affiliations:** University of Massachusetts Chan Medical School, Worcester, Massachusetts, United States; University of Puerto Rico Comprehensive Cancer Center, San Juan, Puerto Rico, United States; University of Massachusetts Chan Medical School, Worcester, Massachusetts, United States

## Abstract

Recruiting participants for qualitative research studies involving older adults and individuals with multiple chronic conditions (MCC) can present unique challenges. This abstract describes the recruitment experiences for two qualitative studies—one on monoclonal gammopathy of undetermined significance (MGUS) and the other on colorectal cancer (CRC) in Puerto Rico. The first study focused on exploring patient experiences with MGUS, a precursor to multiple myeloma, and healthcare providers’ perspectives on managing this condition. Semi-structured interviews were conducted with 14 patients and 8 healthcare providers. The recruitment process involved addressing patients’ uncertainties and concerns about the condition, particularly among older adults. Key themes identified included diagnosis, patient understanding and emotional impact. The second study explored the experiences of CRC patients with MCC in Puerto Rico. Semi-structured interviews were conducted with 9 oncologists and 14 patients. Barriers in care, such as insurance system complexities, limited access to specialized services, and poor communication between patients, caregivers, and providers, were highlighted. A major challenge in this study was recruiting patients who were undergoing cancer treatment. Their ongoing care demands often made participation difficult, as they were overwhelmed by the immediate medical priorities of treatment. Engaging oncologists in interviews and navigating the healthcare infrastructure in Puerto Rico, where access to care is limited, further complicated recruitment efforts. These recruitment experiences underscore the importance of overcoming logistical, social, and emotional barriers to ensure diverse and meaningful participation in qualitative research. The findings highlight the need for tailored approaches to recruitment, particularly for vulnerable populations with complex healthcare needs.

